# Beneficial Endophytic Bacterial Populations Associated With Medicinal Plant *Thymus vulgaris* Alleviate Salt Stress and Confer Resistance to *Fusarium oxysporum*

**DOI:** 10.3389/fpls.2020.00047

**Published:** 2020-02-14

**Authors:** Osama Abdalla Abdelshafy Mohamad, Jin-Biao Ma, Yong-Hong Liu, Daoyuan Zhang, Shao Hua, Shrikant Bhute, Brian P. Hedlund, Wen-Jun Li, Li Li

**Affiliations:** ^1^ CAS Key Laboratory of Biogeography and Bioresource in Arid Land, Xinjiang Institute of Ecology and Geography, Urumqi, China; ^2^ Department of Biological, Marine Sciences, and Environmental Agriculture, Institute for Post Graduate Environmental Studies, Arish University, Al-Arish, Egypt; ^3^ Department of Environmental Protection, Faculty of Environmental Agricultural Sciences, Arish University, Al-Arish, Egypt; ^4^ School of Life Sciences, University of Nevada, Las Vegas, NV, United States; ^5^ State Key Laboratory of Biocontrol and Guangdong Provincial Key Laboratory of Plant Resources, School of Life Sciences, Sun Yat-Sen University, Guangzhou, China

**Keywords:** environmental microbiology, medicinal plants, endophytes, biofertilizer, biocontrol, *Fusarium oxysporum*, *Bacillus subtilis*, *Thymus vulgaris*

## Abstract

As a result of climate change, salinity has become a major abiotic stress that reduces plant growth and crop productivity worldwide. A variety of endophytic bacteria alleviate salt stress; however, their ecology and biotechnological potential has not been fully realized. To address this gap, a collection of 117 endophytic bacteria were isolated from wild populations of the herb *Thymus vulgaris* in Sheikh Zuweid and Rafah of North Sinai Province, Egypt, and identified based on their 16S rRNA gene sequences. The endophytes were highly diverse, including 17 genera and 30 species. The number of bacterial species obtained from root tissues was higher (n = 18) compared to stem (n = 14) and leaf (n = 11) tissue. The endophytic bacteria exhibited several plant growth-promoting activities *in vitro*, including auxin synthesis, diazotrophy, phosphate solubilization, siderophore production, and production of lytic enzymes (i.e., chitinase, cellulase, protease, and lipase). Three endophytes representing *Bacillus* species associated with *T. vulgaris* such as EGY05, EGY21, and EGY25 were selected based on their *ex-situ* activities for growth chamber assays to test for their ability to promote the growth of tomato (*Solanum lycopersicum* L.) under various NaCl concentrations (50–200 mM). All three strains significantly (P < 0.05) promoted the growth of tomato plants under salt stress, compared to uninoculated controls. In addition, inoculated tomato plants by all tested strains decreased (P < 0.05) the activity of antioxidant enzymes (superoxide dismutase, catalase, and peroxidase). Six strains, representing *Bacillus* and *Enterobacter* species EGY01, EGY05, EGY16, EGY21, EGY25, and EGY31 were selected based on *in vitro* antagonistic activity to *F. oxysporum* for pot experiments under salt stress. All tested strains reduced the disease severity index (DSI) of tomato plants at all tested salt concentrations. Gas-chromatography/mass-spectrometry analysis of cell-free extracts of *B. subtilis* (EGY16) showed at least ten compounds were known to have antimicrobial activity, with the major peaks being benzene, 1,3-dimethyl-, p-xylene, dibutyl phthalate, bis (2-ethylhexyl) phthalate, and tetracosane. This study demonstrates that diverse endophytes grow in wild thyme populations and that some are able to alleviate salinity stress and inhibit *F. oxysporum* pathogenesis, making them promising candidates for biofertilizers and biocontrol agents.

## Introduction

Our life is dependent on plants as they produce oxygen and staple foods for domestic animals and humans. The beginning of 21^st^ century is marked by global scarcity of water resources and a 10% annual increase in salinized areas; The Food and Agricultural Organization (FAO) expects an increasing human population to reach 8–9 billion by 2030 (FAO, 2010). As a result of climate change, it has been estimated that nearly 800 million hectares of global land and 32 million hectares of agricultural land has been aﬀected by salinization (El-Beltagy and Madkour, 2012; Ahmad et al., 2015; FAO, 2015). Increased salinity in arid and semiarid regions is a devastating abiotic stress that inhibits normal growth and development of plants, and increases susceptibility of plants to soil-borne pathogens, resulting in considerable reductions in agricultural production and disruption of ecological balance in natural areas (Egamberdieva et al., 2011; Egamberdieva et al., 2013; Li et al., 2018; Mohamad et al., 2018).

In order to enhance agricultural productivity, it is crucial to understand the physiological and biochemical properties evolved by plants to alleviate salt stress (Ahmad et al., 2010a). Plants are equipped with a variety of enzymatic and non-enzymatic mechanisms against salt stress. The antioxidant defense system plays a major role in plant adaptation to salt stress and includes enzymes such as superoxide dismutase (SOD), ascorbate peroxidase (APX), catalase (CAT), and glutathione reductase (GR); non-enzymatic antioxidants include ascorbic acid (ASA) and glutathione (GSH) (Mittler, 2002; Ahmad and Sharma, 2008; Ahmad et al., 2010b; Rasool et al., 2013). Plant cells generate reactive oxygen species (ROS) under normal conditions, but the excessive production of ROS due to osmotic stress causes oxidative damage and affects the functional integrity of cells (Ahmad et al., 2010a; Ahmad et al., 2012c).

Recently, several strategies to alleviate the toxic effects caused by high salinity have been developed, including the use of beneficial microorganisms that act through various direct and indirect mechanisms (Wang et al., 2003; Nia et al., 2012; Ramadoss et al., 2013). Detailed knowledge of interactions between bacteria and plants under salt stress is limited, and the role of microbes in the management of biotic and abiotic stresses is gaining importance for alleviating salinity stress in salt-sensitive crops (Yao et al., 2010; Egamberdieva et al., 2013).

The establishment of successful interactions between microbial symbionts and plants facilitates Induced Systemic Tolerance (IST) response, which has been suggested to be important for eco-friendly and sustainable agriculture (Grover et al., 2011; Beneduzi et al., 2012; Schlaeppi and Bulgarelli, 2015). Endophytes live inside plant tissues without damaging and causing any disease and in many cases, they directly or indirectly improve plant performance under stress by providing them with phytohormones, fixed nitrogen, and nutrients such as soluble iron, potassium, and phosphate (Hayat et al., 2010; Khan et al., 2017; Li et al., 2018).

In the last decade, a vast number of studies have supported the hypothesis that plant growth-promoting bacteria (PGPB) enable plants to maintain productivity under different abiotic stresses by various mechanisms. These bacteria belong to genera such as *Rhizobium*, *Bacillus*, *Pseudomonas*, *Pantoea*, *Paenibacillus*, *Burkholderia*, *Achromobacter*, *Azospirillum*, *Microbacterium*, *Methylobacterium*, *Variovorax*, and *Enterobacter* (Hamdia et al., 2004; Grover et al., 2011; Ahmad et al., 2012a; Bharti et al., 2013; Li et al., 2018). In addition, numerous studies have shown that PGPB enhance root and shoot growth and induce the antioxidant defense system in crops such as lentil (*Lens esculenta*) (Faisal, 2013), pea (*Pisum sativum* L.) (Meena et al., 2015), cucumber (*Cucumis sativus*), (Egamberdieva et al., 2011), rice (*Oryza sativa*) (Yadav et al., 2014) and soybean (*Glycine max*) (Egamberdieva et al., 2016). Therefore, endophytic plant symbionts provide excellent models for understanding the plant-microbe interactions that could potentially be engineered to maintain crop productivity to cope with climate change-induced stresses (Grover et al., 2011).

Medicinal plants have a long history in the development of human culture, and in many developing countries modern medicines are produced from traditional medicinal plants (Tian et al., 2014; Egamberdieva et al., 2017a). However, studies linking the growth of traditional medicinal plants to specific microbial interactions remain in incipient stages (Köberl et al., 2013b). Indeed, the composition of bioactive secondary metabolites synthesized by medicinal plants widely depending on the plant species; these metabolites can strongly aﬀect their association with endophytic microbes (Qi et al., 2012; Chaparro et al., 2014; Cushnie et al., 2014). Hence, understanding the response of microbial communities associated with medicinal plants to alterations in the physicochemical environment of the rhizosphere may provide valuable insights into the ecology of plant-associated bacteria (Köberl et al., 2013a; Egamberdieva and da Silva, 2015; Mohamad et al., 2018). *Thymus* is a traditional medicinal plant in the mint family, *Lamiaceae,* and has a wide distribution in Africa, Europe, and Asia (Hosseinzadeh et al., 2015). *Thymus vulgaris* is used worldwide as an infusion to treat respiratory ailments such as colds, congestion, sore throat, and both upper and lower respiratory infections, diabetes, and intestinal infections and infestations. It also has been described to have antiseptic, antibacterial, and antifungal properties (Ekoh et al., 2014; Prasanth Reddy et al., 2014). However, to date *Thymus* has not been investigated with respect to microbial communities.

Tomato is another economically important crop worldwide. Tomato plants are sensitive to vascular wilt diseases by *Fusarium oxysporum*, particularly under salt stress. (Inami et al., 2014; McGovern, 2015). Symptoms of *F. oxysporum* include yellowing of older leaves and browning of vascular tissues (Heitefuss, 2012). Management of *Fusarium* wilt of tomato is challenging and globally important. Therefore, the objectives of the present study were to 1) isolate and identify beneficial endophytic bacteria associated with wild *T. vulgaris*; 2) evaluate their growth-promoting and salt stress alleviating ability on tomato plants; 3) evaluate their biological control ability against *F. oxysporum in vitro/vivo*; and 4) identify prevalent volatile organic compounds (VOCs) produced by endophytes only in the presence of *F. oxysporum*, which are likely to be effectors of the antifungal properties. To the best of our knowledge, this is the first report on the isolation, identification, and characterization of endophytic bacteria associated with the wild medicinal plant *T. vulgaris*.

## Materials and Methods

### Sample Collection, Plant Material, and Sterilization

Symptom-free *T. vulgaris* plants were collected in the summer of 2016 from their natural arid habitats in North Sinai Province of Egypt. The study sites were within the desert of North Sinai districts Rafah (31°18′16.4″N 34°13′13.3″E) and Sheikh Zuweid (31°05′26.2″N 33°59′48.9″E). *T. vulgaris* populations are abundant at these sites due to their adaptation to poor sandy soil and arid climatic conditions. At each location, three healthy plants located 2–7 m apart were harvested. Whole plants, including root systems (10–15 cm depth), were aseptically harvested, placed in Zip-loc bags, and stored at 4°C during transportation to the laboratory until further processing.

### Isolation of Endophytic Bacteria

Each plant sample was thoroughly washed under tap water and subsequently sterile double distilled water to remove adhering epiphytes and sand debris. Plants were then separated into leaves, stems, and roots, and successively cut into small pieces by using sterile scissors. Approximately 5–7 g of plant tissue was excised from each tissue sample and was surface-sterilized under a laminar airflow cabinet by immersing it sequentially with shaking in 0.1% Tween 20 for a few seconds, 70% ethanol for 3 min, and 5% NaOCl for 5 min, with three rinses with sterile, distilled water between each step of surface sterilization in order to remove the residues and smell of the chemicals used in the surface sterilization protocol (Li et al., 2018). Subsequently, all sterilized samples were cut aseptically into 0.5 cm-long segments using sterile blades or scissors and placed on a piece of sterile filter paper in a laminar air ﬂow chamber for 2–3 h. One gram of each tissue sample was weighed separately and macerated with a sterile mortar and pestle, along with 9 ml of sterile phosphate buﬀered saline (PBS) (20 mM sodium phosphate, 150 mM NaCl, pH 7.4). The homogenate was then transferred to a sterile polypropylene tube and vortexed for 2 min. The tissue homogenate was centrifuged at 2200 × g for 5 min and the supernatant was collected, serially diluted (10^–2^–10^–4^), and then 150 µl aliquots from appropriate dilutions were spread onto ten different isolation media in triplicate (**Table S1**) (Li et al., 2018). The agar plates were sealed with parafilm, incubated at 28 °C, and monitored every five days for microbial growth. Several distinct colony morphologies on each isolation medium were re-streaked for purification on ISP_2_ media. To test the efficiency of plant surface sterilization, controls were also set up by plating 100 µL of sterile ddH_2_O used for the final step of surface sterilization onto the ten media. No microbial growth was detected on the isolation media after 7 days of incubation at 28 °C. This result indicated that the three-step surface sterilization protocol was successful in killing or at least inhibiting the growth of the epiphytic bacteria. Microbial isolates were therefore considered to be true endophytes. All bacterial isolates were stored in 25% glycerol at −80 °C.

### Genotypic Identification

Molecular identification of bacterial isolates was performed after extraction of DNA using Chelex^®^ 100 sodium following the manufacturer’s instructions (SIGMA-ALDRICH). The extracted DNA was dissolved in 20 μL TE buffer and used as the template for PCR. The 16S rRNA gene was amplified using the universal primers 27F (5′-CAGAGTTTGATCCTGGCT-3´) and 1492R (5′-AGGAGGTGATCCAGCCGCA-3´) (Li et al., 2018). Reactions were performed in a Biometra Thermal Cycler. The PCR mixture (25 μL) contained 12.5 μL 2×Taq PCR Master Mix procured from TIANGEN BIOTECH (Beijing, China), 2 μL DNA template (50 ng), and 1 μL of each primer (10 pmol). PCR was performed under the following conditions: initial denaturation step at 95°C for 6 min, followed by 35 cycles of denaturation at 94 °C for 45 (s), annealing at 57 °C for 1 min and extension at 72 °C for 1:30 min, with a final extension step at 72 °C for 10 min. The fragment was sequenced by Shanghai Sangon Biological Engineering Technology & Services Co., Ltd. All 16S rRNA gene sequences were compared with the GenBank database by using the EzBiocloud server (http://www.eztaxon.org) (Chun et al., 2007; Li et al., 2018). All near full-length 16S rRNA gene sequences have been deposited in GenBank under Accession Numbers MH764457–MH764573.

### *In Vitro* Screening for Plant Beneficial Traits

#### Indole Acetic Acid (IAA) Production

Salkowski’s colorimetric method was used to determine the ability of bacterial endophytes to produce indole-3-acetic acid (IAA). Isolates were grown in 25 ml of TYC broth (3 g L^–1^ yeast extract; 5 g L^–1^ tryptone; and 0.872 g L^–1^ CaCl_2_∙2H_2_O) with 0.1% (w/v) L- tryptophan for 2–4 days at 28 ^◦^C at 125 rpm (Egamberdieva and Kucharova, 2009; He et al., 2019). After incubation, the broth was centrifuged at 9,302 × g for 5 min, and 1 ml of supernatant was mixed with Salkowski reagent (2 ml of 0.5 M FeCl_3_, and 98 ml of 35% HClO_4_) (1:1 v/v) and incubated in the dark for 30 min. Development of pink color indicated indole production. Subsequently, results were confirmed by measuring the optical density (OD) at 530 nm and compared comparing with known amounts of IAA using Salkowski reagent and sterile TYC broth with tryptophan as blanks (Li et al., 2018).

### Phosphate Solubilization

All endophytic bacterial isolates were screened for solubilization of inorganic phosphate was evaluated qualitatively on solid Pikovskya’s medium supplemented with Ca_3_(PO_4_)2 (5 g/L) and Bromophenol Blue (0.025 g/L) as described (Paul and Sinha, 2017; Li et al., 2018) with some modifications. After seven days of incubation at 28^◦^C, the formation of yellow halos and/or clearing zones was evaluated. The change of color from blue to yellow or the formation of a clear halo around the colonies was indicative of the utilization of tricalcium phosphate present in the agar medium.

### Nitrogen Fixation Activity

To test nitrogen fixation activity, bacterial isolates were tested on two nitrogen-free media: Ashby’s mannitol agar, composed of (L^−1^) (0.2 g KH_2_PO_4_; 0.2 g MgSO_4_; 0.2 g NaCl; 5.0 g CaCO_3_; 10.0 g mannitol; 0.1 g CaSO_4_; 15.0 g agar; pH 7.0) and NFC medium (0.2 g KH_2_PO_4_; 10.0 g mannitol; 0.2 g MgSO_4_∙7H_2_O; 0.2 g NaCl; 0.2 g CaSO_4_∙2H_2_O; 5.0 g CaCO_3_; 15.0 g agar; pH 7.2) (Liu et al., 2016; Li et al., 2018). Isolates were incubated at 28 ^◦^C for seven days and nitrogen fixation activity was observed based on colony growth on the agar plates.

### Production of Siderophores

Siderophore production was screened based on competition for iron (Fe) between ferric complexes of universal chrome azurol S (CAS) agar media as described (Alexander and Zuberer, 1991; Li et al., 2018). Isolates were incubated at 28 ^◦^C for 5–7 days. Change of the blue color of the medium surrounding the colony and appearance of an orange/purple or purple/red halo zone was scored as positive for production of siderophores (Li et al., 2018).

### Assays for Proteolytic, Lipolytic, Cellulolytic, and Chitinolytic Activity

The bacterial strains were checked for proteolytic activity using the spot inoculation technique on skim milk agar 5% (v/v) medium (Tiru et al., 2013). The skim milk agar plates were incubated for 48 h. Proteolytic activity was identified by the formation of a clear halo around the bacterial colonies due to hydrolysis of skim milk (Li et al., 2018).

Lipolytic activity was assayed using the spot inoculation technique using modified Sierra lipolysis agar supplemented with beef extract (3 g L^–1^) and ferrous citrate (0.2 g L^–1^). After autoclaving, 50 mL of Victoria Blue B solution (0.1 g per 150 mL) and 10 mL of Tween 80 was added to the medium. After 5–6 days of incubation, white calcium precipitates around bacterial colonies indicated a positive reaction (Li et al., 2018).

Cellulolytic activity was assayed with modified DSMZ medium 65 (http://www.dsmz.de/microorganisms/medium/pdf/DSMZ_Medium65.pdf) without CaCO_3_ and supplemented with carboxymethyl cellulose (5 g L^–1^; Sigma) in place of glucose by using the spot inoculation technique. After incubation for 5-6 days, plates were stained with a Congo red solution and destained with a NaCl solution (Teather and Wood, 1982; Li et al., 2018; Mohamad et al., 2018). A clear or lightly colored halo around the colonies indicated a positive reaction.

Colloidal chitin was prepared from commercial chitin by the method of Agrawal and Kotasthane (Agrawal and Kotasthane, 2012; Mohamad et al., 2018). Chitinase detection medium consisted of (L^−1^) 0.3 g of MgSO_4_.7H_2_O, 3.0 g of (NH_4_)_2_SO_4_, 2.0 g of KH_2_PO_4_, 1.0 g of citric acid monohydrate, 15 g of agar, 200 μL of Tween-80, 4.5 g of colloidal chitin and 0.15 g of bromocresol purple per liter and then autoclaved at 121^◦^C for 15 min. Chitinolytic activity was assessed using the spot inoculation technique by observation of colored zones around colonies. All screening experiments for plant beneficial traits were performed twice with three replicates for each individual isolate.

### Plant Growth-Promoting Activity in Saline Soil

Pot experiments were carried out in a growth chamber to investigate the symbiotic effects of bacterial isolates on plant growth in non-saline and saline soils. Tomato seeds (*Solanum lycopersicum*. cv. Fuji Pink) were surface-sterilized by immersion in sodium hypochlorite (2% v/v) for 2 min and subsequently rinsed five times with ion-free distilled water (Cao et al., 2004). Sterilized seeds were germinated on a wet filter placed in a Petri dish (9 cm diameter). The Petri dishes were covered with a polyethylene sheet to prevent evaporation and kept in the plant growth chamber at 25^◦^C for 4–5 days.

Endophytic strains were grown overnight in ISP_2_ broth, and the cell suspension was centrifuged. The cell pellets were resuspended at a final concentration of 10^8^ CFU/ml with phosphate-buffered saline PBS and adjusted by using Densicheck plus (Biomerieux, USA) (Errakhi et al., 2007; Egamberdieva et al., 2011). Tomato seedlings were inoculated by soaking roots for 15 min in a 1 mL solution each bacterial suspension and shaking gently with sterile forceps (Botta et al., 2013). The inoculated seedlings were aseptically transplanted into plastic pots (12 cm high × 10 cm in diameter) filled with autoclaved compost: sand: perlite: peat (1:1:1:1, v/v/v/v) and placed in a growth chamber at 25 ± 2°C and a 14-h photoperiod. After 3 days, 10 ml of the bacterial suspension (10^8^ CFU/mL) was added near the root zone and salinity was increased gradually by applying a sodium chloride solution to each pot on alternative days to avoid osmotic shock to reach final salt concentrations of 50, 100, 150, and 200 mM and the desired salt concentrations of 50, 100, 150, and 200 mM were achieved after 2, 4, 6, and 8 days, respectively (Nejad and Johnson, 2000; Chatterjee et al., 2017). Parallel controls were maintained by cultivating tomato plants with autoclaved compost without inoculation of endophytes and irrigating with tap water. Each treatment contained three pots and each pot including four tomato seedlings. After 8 weeks, the tomato plants were harvested for growth and antioxidant enzyme assays. Plants were uprooted and washed to remove adhering peat. Shoot and root length and fresh weight were recorded.

### Plant-Microbe Response Defense Under Saline Condition

#### Determination of Antioxidant Enzymatic Activity

Plant extracts were prepared from tomato leaves after 45 days and antioxidant enzymes were assayed as described by Ahmad et al. (2015). Brieﬂy, fresh tomato leaves were ground using liquid nitrogen and the ground leaf samples were stored at –80 ^◦^C. The ground leaf samples (approximately ∼1 g) were homogenized on ice using 10 mL of 50 mM phosphate buﬀer (pH 7.8) and then incubated for 10 min at 4 ^◦^C. Subsequently, the homogenate was filtered using Advantech Qualitative Filter Papers (110 mm) and centrifuged at 4,000 × g for 15 min at 4 ^◦^C. The supernatant was used for the determination of enzyme activities. The activities of superoxide dismutase (SOD, EC 1.15.1.1), catalase (CAT) (EC 1.11.1.6), and peroxidase (POD, EC 1.11.1.7), were measured using assay kits (kit Numbers. A001-1, A007-1, A084-3, respectively; Nanjing Jiancheng Bioengineering Institute, China), following the manufacturer’s instructions (http://elder.njjcbio.com/index_en.php) (Liang et al., 2017). This experiment was conducted in triplicate.

### Photosynthetic Pigments

Chlorophyll content was quantified by using the Leaf Chlorophyll Meter (SPAD 502 Plus). Readings were taken on the uppermost fully expanded leaf with a visible collar during vegetative growth, and from the ear leaf (20 leaves per treatment) as suggested (Dwyer et al., 1995).

### *In Vitro* Screening for Antifungal Activities on Solid Medium

Antifungal activity was screened *in vitro* against the following pathogenic microorganisms *F. oxysporum* f. sp. (F1), *Fulvia fulva* (Cooke) Cif (F2), and *Alternaria solani* Sorauer (F3) (**Table S2**). Brieﬂy, fungal strains were grown in potato dextrose agar (PDA) plates for 6 days and the mycelial disc (5 mm) was transplanted into the center of a fresh PDA plate. Bacterial cultures pre-grown in ISP_2_ media for 3 days were symmetrically spotted onto the four corners of the plate, 2.5 cm from the plate periphery. All plates were wrapped with parafilm and incubated at 26 ± 2 ^◦^C for 3 days and observed for the inhibition of the pathogen. Activity was quantified by measuring the zone of inhibition of the pathogen’s growth (Velusamy et al., 2006; Kilani-Feki et al., 2011) and the percent inhibition was calculated using the following formula (Yasmin et al., 2016; Mohamad et al., 2018).

$$\text{Percent inhibition }(\%)=\frac{{\text{F}}_{\text{cd}}-{\text{T}}_{\text{fcd}}}{{\text{F}}_{\text{cd}}-{\text{F}}_{0}}\times 100$$

where F_cd_ is the fungal colony diameter on the control PDA base plate, T_fcd_ is the fungal colony diameter on the treatment PDA base plate, and F_0_ is the diameter of the test fungus agar discs (approximately 5 mm). This experiment was conducted twice in triplicate.

### Biological Control of Tomato Root Rot by Endophytic Bacterial Strains *In Vivo*

Bacterial isolates with antagonistic activity against at least two of the tested fungal pathogens were tested for their ability to control tomato root rot caused by *F. oxysporum* in saline soils. Tomato seedlings were aseptically planted in plastic pots filled with non-saline and saline soil as described above. *F. oxysporum* from 5-day-old potato dextrose broth was filtered with 6 layers of sterile gauze to remove mycelia and the spores were washed with sterile water and diluted to a concentration of 10^7^ conidia/ml (Biomerieux, USA) (Fakhouri and Buchenauer, 2003). Bacterial suspensions were prepared as described above. The treatments were: (i) control, without pathogen and bacteria; (ii) control with pathogen only; and (iii) pathogen and bacteria. Plants at the four-true-leaf stage were wound-inoculated with spores by puncturing the stem at the first internode above the soil line with a 22-gauge needle by sterile syringe (Hanson, 2000; Fakhouri and Buchenauer, 2003; Chen et al., 2014; Mohamad et al., 2018). 10 mL of the bacterial suspension (10^8^ CFU/mL) was applied near the root zone as described above 3 days after wounding.

Disease symptoms were recorded for 45 days following pathogen challenge, including leaf yellowing and chlorosis. Disease severity was classified into six grades (i.e., grades 0, 1, 2, 3, 4, and 5) according to the visible symptoms on the cotyledons and true leaves (Amini, 2009; Zheng et al., 2013; Mohamad et al., 2018). The scale estimated the percentage of affected leaves using five main categories or quarters (≤35, 35–55, 55–65, 65–85 and 85–100%) with five values for each category. The disease index (DI) was calculated according to the following formula: DI = [(∑ disease grades × number of infected)/(total checked plants ×5)] × 100 (Zheng et al., 2013; Mohamad et al., 2018). The disease index (DI) represents a comprehensive and objective measure of plant health, with high DI values corresponding to serious infections. In this experiment, each treatment contained three pots and each pot including four tomato seedlings. Average disease index and standard deviation were calculated for each isolate at each salt concentration; statistical analysis (ANOVA followed by Tukey’s Honest Significant Difference post-hoc test) was conducted to identify significant differences.

### Extraction of Metabolites

The antibiosis experiment was carried out by co-cultivation of strain EGY16 with *F. oxysporum* in 500 mL^–1^ of broth medium at 28^◦^C for 12 days with agitation at 150 rpm in triplicate. Cells were collected by centrifugation at 5,000 × g for 10 min. The cell-free supernatant was mixed with an equal volume (1:1) of ethyl acetate by vigorous shaking for 60 min and allowed to settle. The organic solvent phase was evaporated at 40^◦^C under vacuum, using a rotary evaporator (IKA, HB10 basic). The ethyl acetate extract was dissolved in 5 mL of Tris–Cl buffer (0.02 M, pH 7.0) and used for gas-chromatography/mass spectrometry (GC-MS) (Mohamad et al., 2018).

### Identification of Metabolites

GC-MS analysis of the cell-free extracts was performed using a gas chromatograph (Model 7890A, Agilent, Palo Alto, CA, USA) equipped with a split-splitless injector, an Agilent model 7693 autosampler, and an Agilent HP-5MS fused silica column (5% Phenyl-methylpolysiloxane, 30 m length, 0.25 mm I.D., film thickness 0.25 mm). Injecting volume was 1 µL, and the GC conditions included programmed heating from 50 to 300 °C at 10 °C/min, followed by 10 min at 300 °C. The injector was maintained at 280 °C. Helium was the carrier gas, at 1.0 mL min^−1^, and the split mode was 5:1. The GC was fitted with a quadrupole mass spectrometer with an Agilent model 5975 detector. The MS conditions were as follows: ionization energy, 70 eV; electronic impact ion source temperature, 230 °C; quadrupole temperature, 150 °C; scan rate, 3.2 scans/s; mass range, 50–1000 u. The compounds were identified based on the match with their mass spectra and retention indices with the NIST/Wiley 275 library (Wiley, New York). The Relative abundance of each feature was calculated from Total Ion Chromatogram (TIC) computationally (Mohamad et al., 2018).

### Intelligent Live Digital Imaging of Antibiosis Endophytes Strains and *F. oxysporum*

The morphological response of selected bacterial strains *Bacillus sonorensis* (EGY05), *Bacillus subtilis* subsp. subtilis (EGY01), *Bacillus tequilensis* (EGY21), *Bacillus mojavensis* (EGY25), *Enterobacter xiangfangensis* (EGY31), and *Bacillus subtilis* subsp.inaquosorum (EGY16) had demonstrated antagonism to *F. oxysporum in vitro* and *in vivo* were observed under a laser microscope (Olympus SZX2-ILLT, Japan) at different magnifications.

Six selected antagonistic bacterial strains were incubated on ISP_2_ medium. *F. oxysporum* was grown on ISP_2_ medium. A six-day-old mycelial disc (5 mm) was placed at the center of a 7 cm modified culture ISP_2_ plate. The bacteria were placed at four corners on the bacterial lawn at equidistant points 2.5 cm from the plate periphery. All plates were wrapped with parafilm, incubated at 28 ± 2^◦^C for 6 days, and observed for the inhibition of the pathogen. Plates with pathogenic fungi alone served as control (Mohamad et al., 2018).

### Statistical Analysis

The data represent mean of at least 10–12 replicates ± standard error (SE). One-way ANOVA was used to compare the means of root length, root fresh weight, shoot length, shoot fresh weight, SOD, POD, CAT, and Chlorophyll content for each salt concentration separately and Tukey’s HSD post-hoc test used for multiple comparisons at alpha level = 0.05. Statistical analyses were conducted in R (R Core Team). (Pinheiro et al., 2018). R: A language and environment for statistical computing. R Foundation for Statistical Computing, Vienna, Austria. URL https://www.R-project.org/.

## Results

### Isolation and Identification of Endophytic Bacteria Associated With *T. vulgaris*

**Figure 1:**
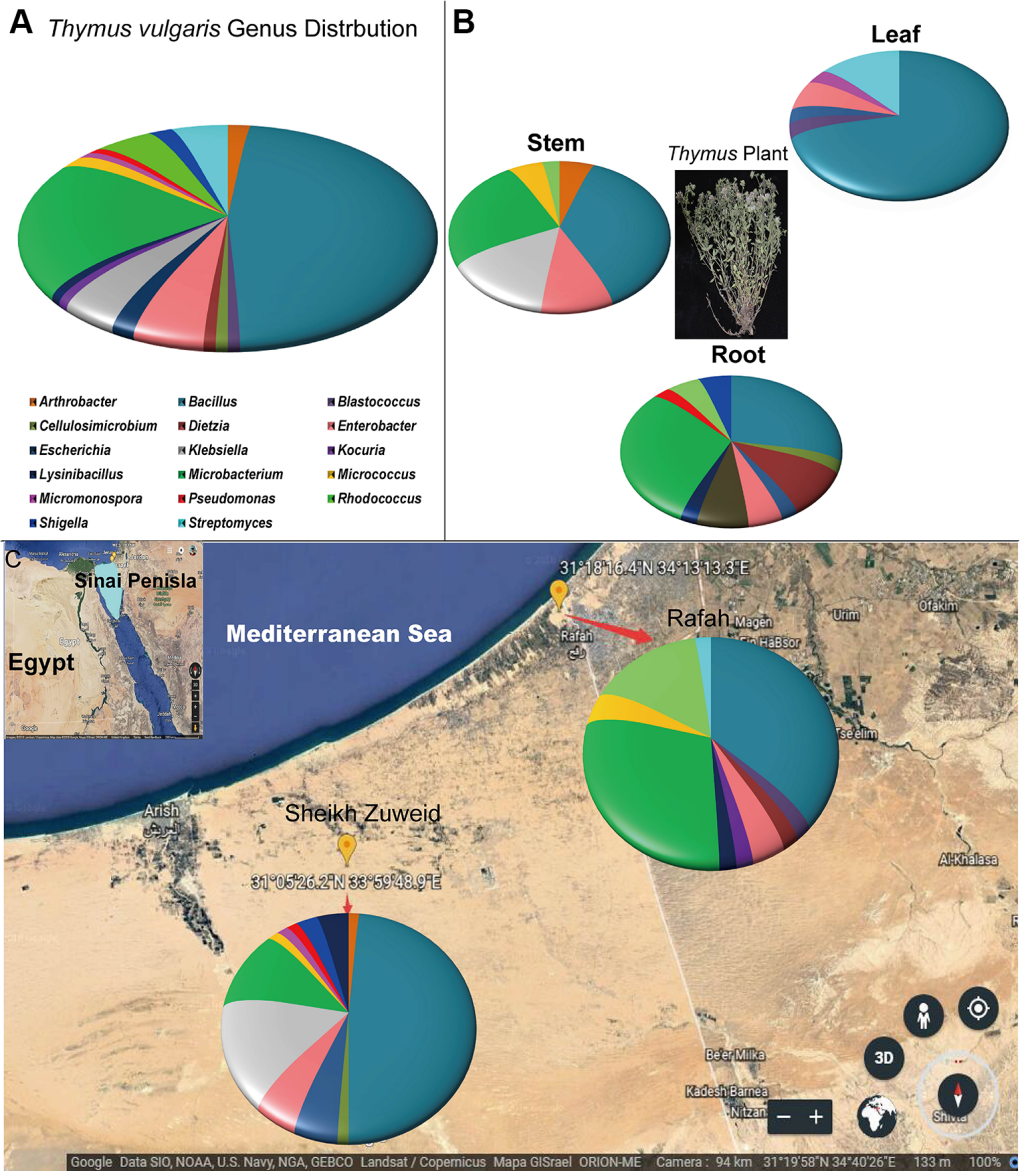
The geographic distribution and identity of 117 endophytes from *Thymus vulgaris* based on 16S rRNA gene sequences. **(A)** A summary of genera presents at all sites. **(B)** Genus assignments for isolates from different tissues, showing high diversity in the root and low diversity in the leaf. **(C)** Genus assignments according to location, showing similar diversity of isolates at the two sites.

Bacterial endophytes were isolated from roots, stems, and leaves of six wild *T. vulgaris* plants collected from two different locations in North Sinai, Egypt. A total 117 strains were isolated based on unique colony morphologies on ten different isolation media. Based on nucleotide identity and phylogenetic analysis of near-complete 16S rRNA gene sequences, the isolates belonged to 17 different genera (**Figure 1A**) and 30 species (**Figure S1**). Most isolates belonged to *Bacillus* (47%), followed by *Microbacterium* (20%); *Enterobacter* (5.5%); *Streptomyces*, *Rhodococcus*, and *Klebsiella* (4.6% each); *Arthrobacter*, *Escherichia*, *Micrococcus*, and *Shigella* (1.85% each); and *Kocuria*, *Dietzia*, *Lysinibacillus*, *Blastococcus*, *Cellulosimicrobium*, *Pseudomonas*, and *Micromonospora* (0.93% each).

The diversity of the isolates varied based on the plant tissue. The highest number of distinct bacterial species was recovered from root tissue (n = 18), compared to stems (n = 14) and leaves (n = 11). Almost all isolates were belonged to genus *Bacillus* or to various genera within the phylum *Actinobacteria*. *Pseudomonas*, *Cellulosimicrobium*, *Dietzia*, *Shigella*, and *Lysinibacillus* species were only associated with root tissues. *Arthrobacter* and *Kocuria* species were only associated with stem tissues. *Micrococcus* species were only isolated from leaf tissue (**Figure 1B**).

The diversity of endophyte isolates was similar at the Sheikh Zuweid (n = 22 species) and Rafah (n = 17 species) sampling sites; however, the species composition at each site was distinct. In particular, *Bacillus*, *Cellulosimicrobium*, *Enterobacter*, *Klebsiella, Micrococcus, Micromonospora, Pseudomonas,* and *Shigella* species were only isolated from the Sheikh Zuweid site and *Blastococcus*, *Dietzia*, *Kocuria*, *Lysinibacillus*, *Micrococcus*, and *Rhodococcus* species were only isolated from the Rafah site (**Figure 1C**). Meanwhile, the highest number of endophytic species was isolated on M1 medium (n = 13), including members of the genera *Bacillus*, *Microbacterium*, *Cellulosimicrobium*, *Enterobacter*, *Klebsiella*, *Streptomyces*, and *Micromonospora*. The lowest number of species were obtained on M3 and M10 (n = 7 each) (**Figure S2**).

### Beneficial Plant Traits of Endophytic Bacteria

**Figure 2:**
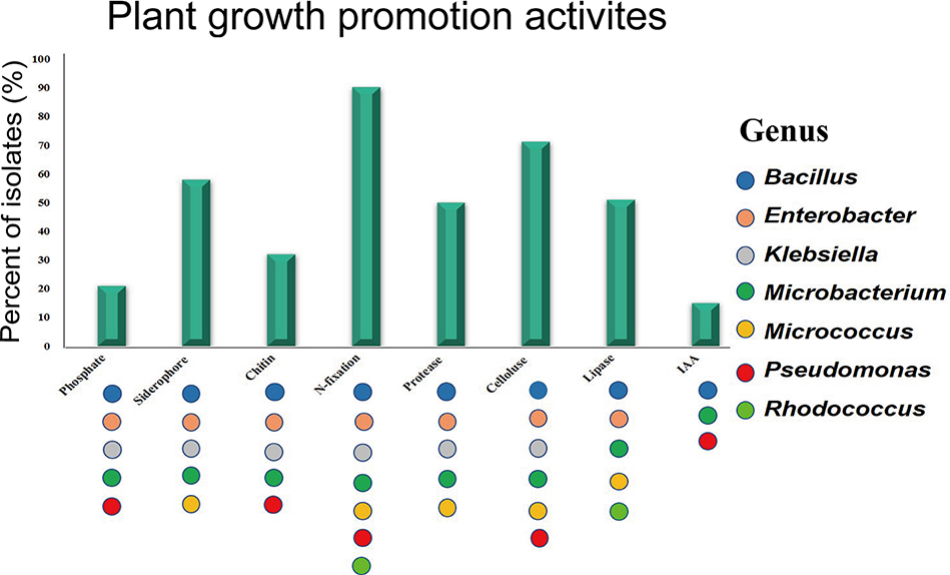
Plant growth promotion activities *in vitro*. Colored dots represent activities observed in at least one isolate from the genus.

All isolates were screened for multiple beneficial traits *in vitro* (**Figure S3**). The majority of isolates was able to fix nitrogen (84%) based on growth on both NFC and Ashby’s media. Many isolates produced siderophores (54%), solubilized phosphate (20%), and synthesized IAA (15%). Strains have more beneficial traits belonged to the genera *Bacillus*, *Enterobacter*, *Pseudomonas*, *Klebsiella*, and *Microbacterium* (**Figure 2**). In addition, all the isolates were screened for the presence of digestive enzymes that may be involved in lysis of fungal pathogens. Most of the endophytic strains produced one or more hydrolytic enzymes: cellulase (66%), lipase (47%), protease (46%), and chitinase (30%) (**Figure 2**). The endophytes producing at least three digestive enzymes belonged to the genera *Bacillus*, *Enterobacter*, and *Micrococcus*.

### Antifungal Activity

**Figure 3:**
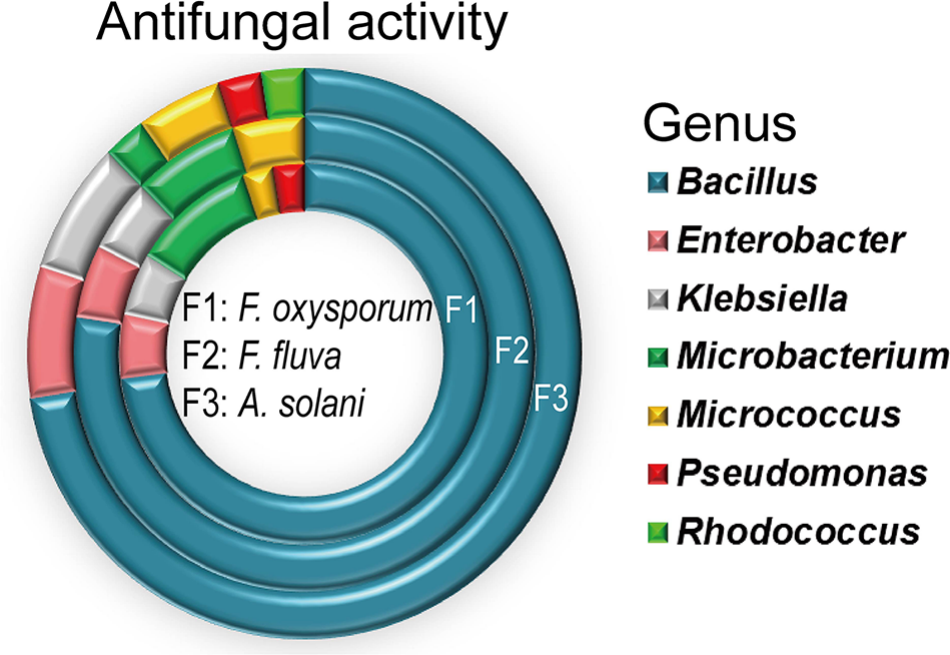
Antimicrobial activity of *Bacillus* species against three pathogenic fungi, shown by number of isolates in each genus with antimicrobial activity. All experiments were performed twice with three replicates for each individual isolate.

All isolates were individually tested against three common tomato pathogens *in vitro*. Endophytes belonging to 7 genera and 12 species were antagonistic to all the tomato pathogens (**Figure 3**). *Bacillus* genus showed the highest antagonistic activity. In addition, the ability to inhibit the growth of the tested fungi varied by the percentage of inhibition, ranging from 40 to 77%. *Enterobacter xiangfangensis* strain EGY31*, Bacillus sonorensis* strain EGY11, and *Bacillus subtilis* subsp. *inaquosorum* strain EGY15 showed the largest zones of inhibition against *F. oxysporum* f. sp. (F1; 70%), *F. fulva* (F2; 71%), and *A. solani* (F3; 77%), respectively.

### Stimulation of Tomato Growth by Endophytes Under Salt Stress

**Figure 4:**
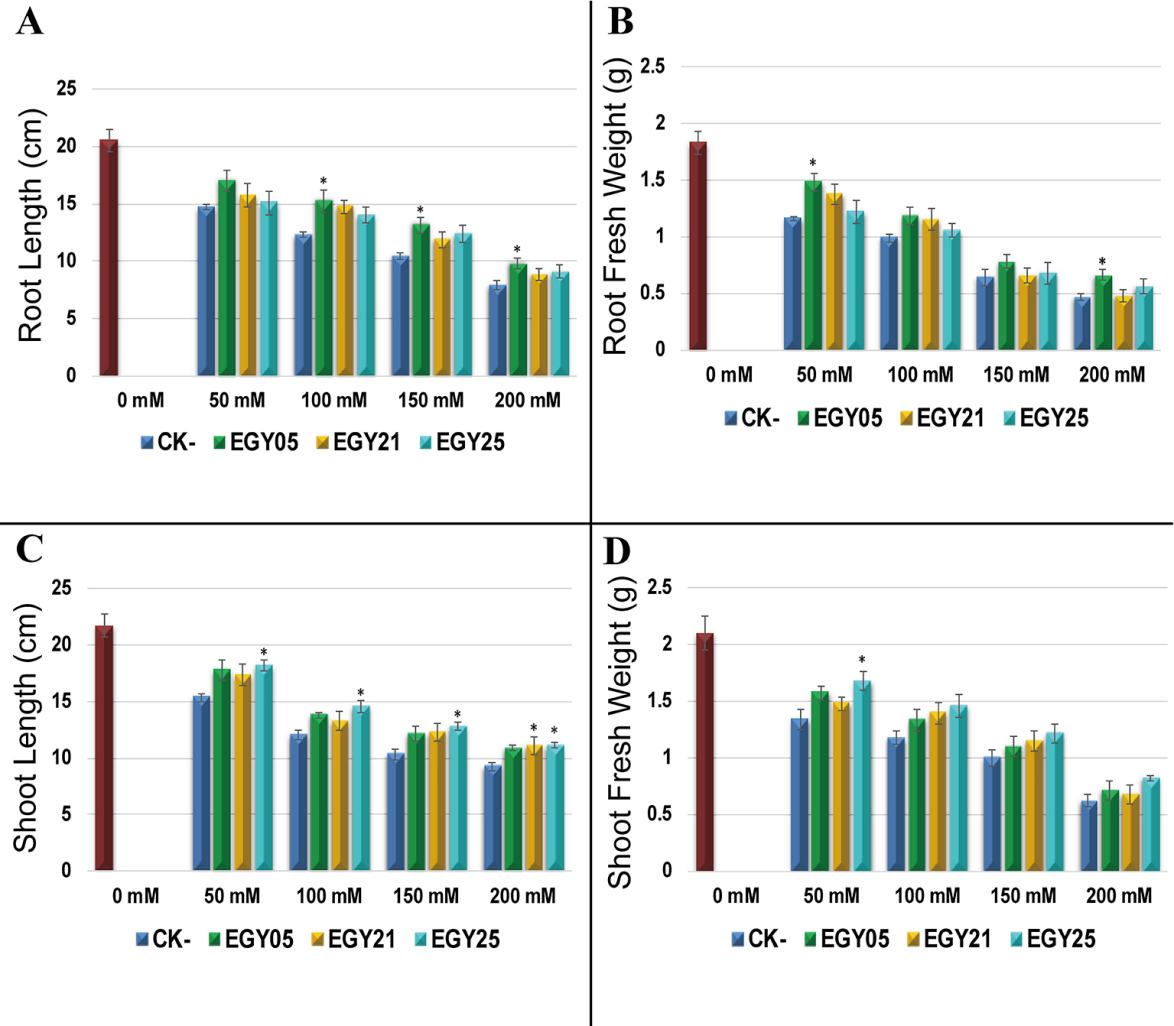
The response of tomato (*Solanum lycopersicum* L.) 55 days after inoculation with the selected endophytic bacteria under salt stress, compared with an uninoculated control plant. In this experiment the data represent mean of at least 10–12 replicates ± standard error (SE). Column marked by (“*”) indicate significant differences based on One-way ANOVA, followed by Tukey’s HSD post-hoc test for multiple comparisons at alpha level = 0.05.

To test whether the endophytes promote plant growth, three bacterial strains, *Bacillus sonorensis* (EGY05), *Bacillus tequilensis* (EGY21), and *Bacillus mojavensis* (EGY25), that were positive for at least four plant-beneficial traits were selected to test their plant growth stimulation properties in pot experiments with tomato plants under salt stress. Shoot and root length and weight decreased with increasing the salt concentrations from 50 mM to 200 mM (**Figure 4**). Tissues from plants inoculated with each of the endophytes were generally larger (P < 0.05) than uninoculated controls but the precise pattern of growth stimulation varied by plant tissue and salinity (**Figure S4**). Strain EGY05 showed the strongest stimulation of root growth, increasing both root length and root fresh weight significantly (P < 0.05) at most concentrations, compared to the un-inoculated controls (**Figure 4A**). In particular, inoculation with strain EGY05 increased the root length by 15.9, 24.4, 25.4, and 23.8% under 50, 100, 150, and 200 mM NaCl treatments, respectively, compared to the uninoculated controls. Moreover, strain EGY05 increased the root fresh weight (**Figure S5**) by 27.6, 19.2, 21.9, and 40.2% under 50, 100, 150, and 200 mM NaCl treatments, respectively (**Figure 4B**).

For shoot fresh weight and shoot length, strain EGY25 showed the strongest stimulatory effects at all salinities tested. Strain EGY25 increased shoot length significantly (P < 0.05) by 18.5, 20.3, 23.3, and 20.0% under 50, 100, 150, and 200 mM of NaCl treatment, respectively, compared to the uninoculated control (**Figure 4C**). It also increased shoot fresh weight over the controls by 25.4, 24. 8, 21.0, and 32.3% under 50, 100, 150, and 200 mM of NaCl treatment; however, this effect was only significant at 50 mM NaCl (**Figure 4D**).

### Effect of Endophytes on Antioxidant Enzyme Activity and Chlorophyll Content Under Salt Stress

**Figure 5:**
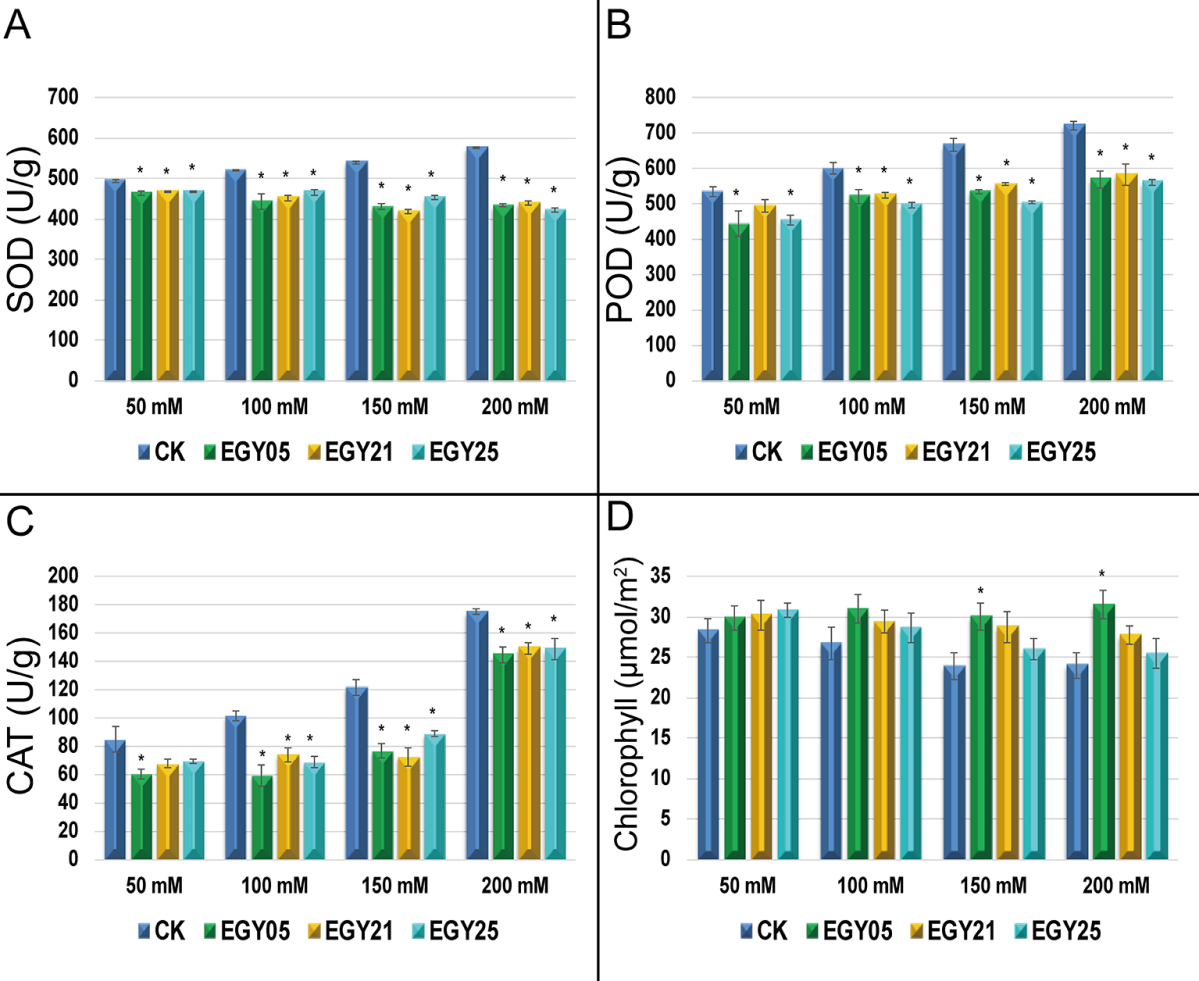
Activities of antioxidant enzymes under salt stress in the presence and absence of selected endophytic bacteria isolated from *Thymus vulgaris*. **(A)** Superoxide dismutase (SOD);**(B)** peroxidase (POD); **(C)** catalase (CAT); and **(D)** photosynthetic pigments. This experiment was conducted twice in triplicate and the mean of at least 3 replicates ± standard error (SE) were calculated for SOD, POD, and CAT. But for photosynthetic pigments (n = 20). Column marked by (“*”) indicate significant differences based on One-way ANOVA, followed by Tukey’s HSD post-hoc test for multiple comparisons at alpha level = 0.05.

The activity of the antioxidant enzymes superoxide dismutase (SOD), catalase (CAT), and peroxidase (POD) were measured in the tomato tissues (**Figure 5**). Salinity stress caused a significant increase in activities of all antioxidant enzymes in this study (P < 0.05). However, each of the three endophytes significantly (P < 0.05) decreased the activity of SOD, CAT, and POD under most conditions. For example, plants inoculated with strain EGY05 showed 6.6, and 17.7, 25.7, and 33.0% decreases in superoxide (SOD) activity at 50, 100, 150, and 200 mM NaCl, respectively, in comparison to the uninoculated control plants (**Figure 5A**). The same strain reduced POD activity by 20.3, 14.9, 32.5, and 28.7% at 50, 100, 150, and 200 mM NaCl (**Figure 5B**). Catalase activity (CAT) (**Figure 5C**) showed a similar pattern where inoculation with strain EGY05 significantly reduced the activity at 50, 100, 150, and 200 mM salt to 40.1, 71.5, 58.3, and 21.4%, respectively, as compared to the control. Salinity also affected chlorophyll content, but inoculation with the endophytes increased chlorophyll content under all conditions; however, this effect was only significant (P < 0.05) for strain EGY05 at 150 and 200 mM NaCl (25.4, and 31.5% increase, respectively) (**Figure 5D**).

### *In Vivo* Biological Control of *F. oxysporum* Under Salt Stress

To evaluate the role of endophytic bacteria on resistance to pathogens *in vivo*, six strains, *Bacillus sonorensis* (EGY05), *Bacillus subtilis* subsp. *subtilis* (EGY01), *Bacillus tequilensis* (EGY21), *Bacillus mojavensis* (EGY25), *Enterobacter xiangfangensis* (EGY31), and *Bacillus subtilis* subsp. *inaquosorum* (EGY16), were selected for their high antagonistic activity to *F. oxysporum* in pot experiments under salt stress.

**Figure 6:**
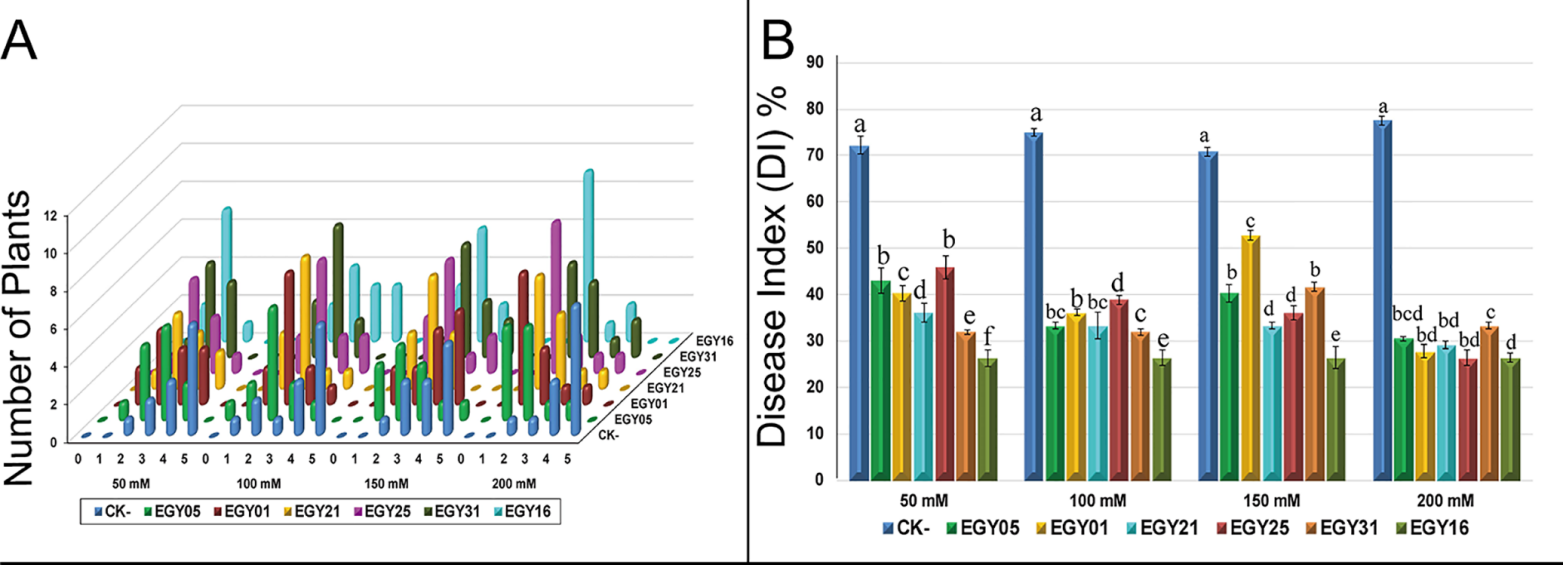
Effect of endophytic isolates on disease grades and disease index of *Solanum lycopersicum L*. plants to *Fusarium oxysporum* over eight weeks under salt stress. **(A)** Signs for individual plants rated from “0” to “5” (0: no signs, 1: ≤35, 2: 35–55, 3: 55–65, 4: 65–85, 5: 85– 100%); **(B)** Disease index of tomato plants with and without endophytes under salt stress. Bar height represents the mean disease index for each isolate at each salt concentration, whiskers represent standard deviation. Shared letters between two isolates at a given salt concentration indicate no significant difference in disease index as calculated using ANOVA followed by Tukey’s HSD post-hoc test, at alpha level = 0.05.

Plant disease signs under salt stress developed within ten days of inoculation. The symptoms included yellowing of leaves, and leaf chlorosis, which began with older leaves and progressed to younger leaves and continually increased throughout the experiment over an 8-week period. The distribution of disease grades varied dramatically at different salt concentrations compared to uninoculated controls (**Figure 6A**). Severe signs reaching 76.4% (DI) were seen on the leaves of the tomato plants challenged with *F. oxysporum* in the absence of endophytes at different salt concentrations (**Figure 6B**). All selected antagonistic bacterial isolates were able to control tomato disease caused by *F. oxysporum* under NaCl treatment, with statistically significant (P < 0.05) effects, at different salt concentrations, in comparison to the pathogen-infected not treated controls. The results showed a dramatic decrease in disease symptoms and enabled plants to survive at different levels of salt stress (**Figures 6A, B**).

### Detection of Bioactive Compounds by GC-MS Analysis

**Figure 7:**
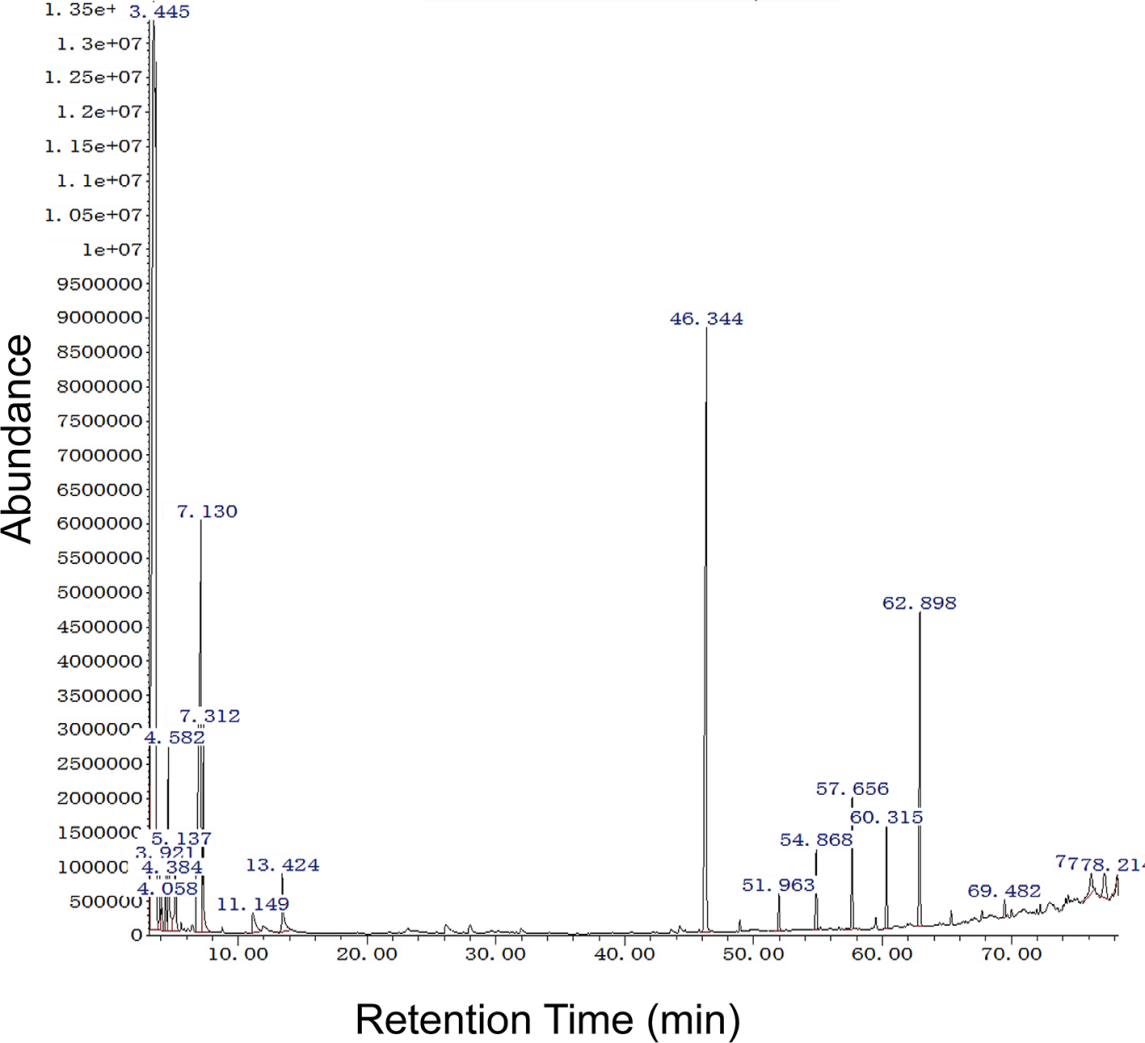
GC-MS analysis of potential bioactive compounds in crude extract of the mixture of EGY16 and *F. oxysporum* at pH 7. Six major features were obtained at RT 3.444, 4.582, 7.131, 7.312, 46.345, 60.315, and 62.898, suggesting the presence of acetic acid, butyl ester; benzene, 1,3-dimethyl-; propane, 1-ethoxy-; propane, 1-ethoxy-; dibutyl phthalate; tetracosane; and bis (2-ethylhexyl) phthalate. This experiment was conducted in triplicate.

To evaluate the volatile compounds produced by the most bioactive strain, *B. subtilis* EGY16, and *F. oxysporum* were grown in co-culture. GC-MS analysis of ethyl acetate crude extracts showed 20 compounds (**Table S3**) at pH 7 and all features were tentatively identified based on a comparison of spectra available through the National Institute of Standards and Technology (NIST) database. Six major features were obtained at RT 3.444, 4.582, 7.131, 7.312, 46.345, 60.315, and 62.898, suggesting the presence of acetic acid, butyl ester; benzene, 1,3-dimethyl-; propane, 1-ethoxy-; propane, 1-ethoxy-; dibutyl phthalate; tetracosane; and bis (2-ethylhexyl) phthalate. In addition, several minor peaks were present, including 2,2-Dimethylthiirane; 2-Methylpropanoic acid, TMS derivative; benzene, 1,3-dimethyl-; p-Xylene; hexane-1,3,4-triol, 3,5-dimethyl-; phenylethyl alcohol; heneicosane; nonadecane; tricosane; decanedioic acid, bis (2-ethylhex; Propenone, 1-(3-bromophenyl)-3; heneicosane, 11-cyclopentyl-; and cyclohexanecarboxylic acid, 2 (**Figure 7**).

### Defense Response of Endophytic Strains to *F. oxysporum via* Laser Microscopy

**Figure 8:**
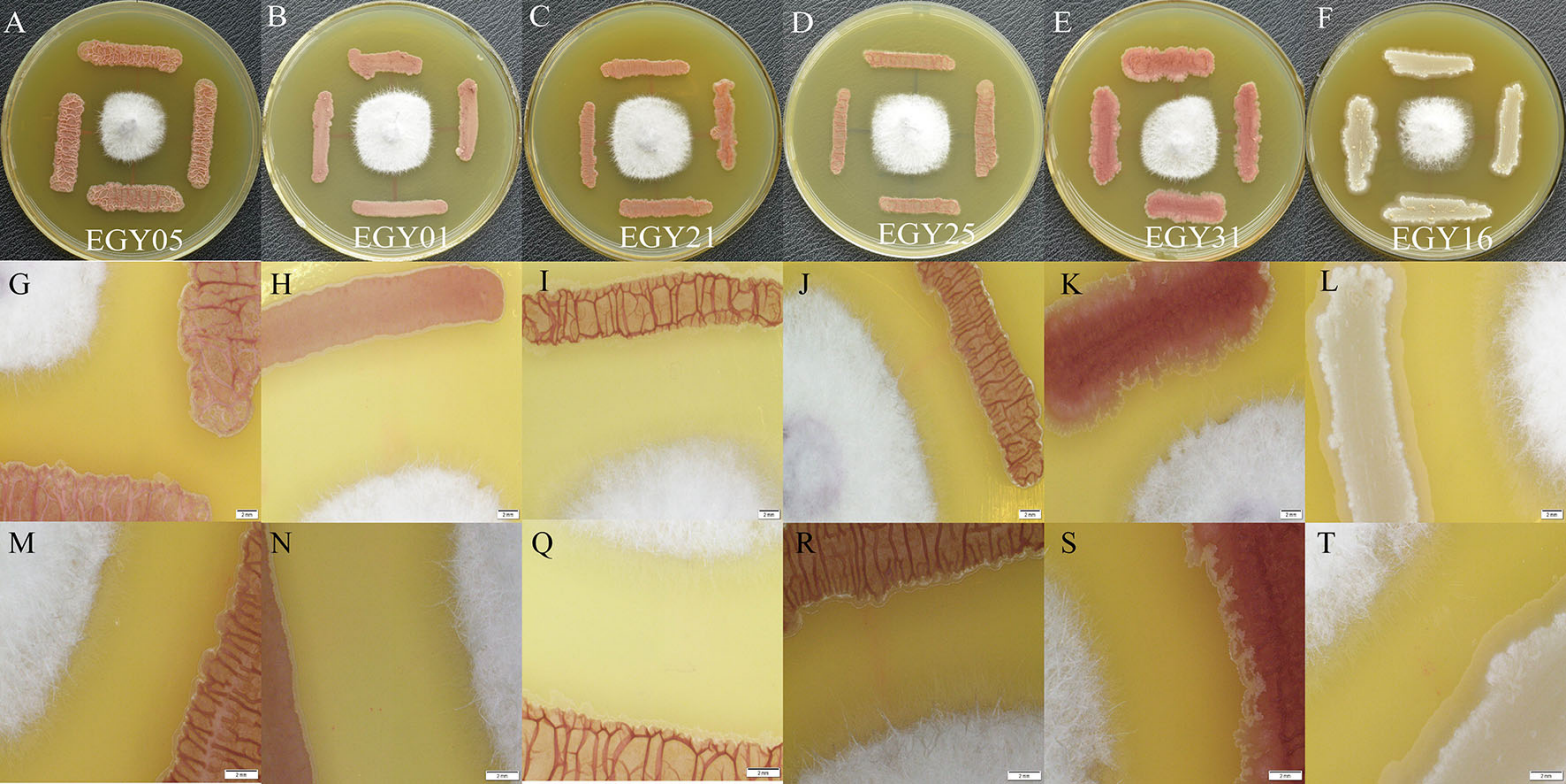
Intelligent live digital imaging of the response of endophytic strains EGY05, EGY01, EGY21, EGY25, EGY31, and EGY16 to *Fusarium oxysporum* under a laser microscope **(A**–**F)**. *In vitro* evaluation of antagonistic activity of tested strains againist strain *F. oxysporum* **(G**–**L)**. Response of tested strains to *F. oxysporum* under a laser microscope at 0.63X magnification **(M**, **N**, **Q**–**T)**. Response of tested strains to *F. oxysporum* under a laser microscope at 1X magnification.

The results showed that *F. oxysporum* could not grow normally after 5 days of incubation with antagonistic strains (**Figures 8A–F**). The morphological response of tested strains EGY05, EGY01, EGY21, EGY25, EGY31, and EGY16 to *F. oxysporum* under a laser microscope were observed at magnifications of 0.63X (**Figures 8G–L**) and 1X (**Figures 8M–T**). The microscopic characteristics of tested strains based on laser microscopy at 0.63X showed that the endophytic strains were able to control the growth of the fungal mycelium after 5 days of cultivation, and at 1X, a white powder appeared around bacterial cells. Thus, we hypothesize that these antagonistic strains may secrete antifungal compounds (**Figures 8M–T**).

## Discussion

As a result of climate change, salinity has become one of the major abiotic stresses that reduce plant growth and crop productivity worldwide. A better understanding of the impacts of endophytic bacteria on plant health under conditions of salinity stress can lead to insights into improved cultivation techniques under stressful conditions.

Thyme has pharmacological properties such as anti-inflammatory, anti-bacterial, anti-viral, antioxidant, and insecticidal activities (Prasanth Reddy et al., 2014; Mandal and DebMandal, 2016). However, to date, *Thymus* has not investigated with respect to microbial communities. Thus, in this study, a collection of 117 endophytic strains associated with roots, leaves, and stems of the medicinal plant *T. vulgaris* was obtained from two different wild populations in North Sinai, Egypt. Isolates were identified by 16S rRNA gene sequencing. The isolates belonged to 17 genera and 30 species mainly belonging to the phyla *Firmicutes*, *Actinobacteria*, and *Proteobacteria*. These results confirm the rich microbial diversity in plants grown in arid lands, in agreement with previous studies done by our group (Liu et al., 2016; Liu et al., 2017; Li et al., 2018). In addition, the bacterial taxa observed were similar to endophytes observed using cultivation-independent techniques (Qin et al., 2012; Kaplan et al., 2013; Jin et al., 2014; Egamberdieva et al., 2017b). The dominant bacterial genus was *Bacillus,* which is known for its beneficial effects on plant growth and health (Radhakrishnan et al., 2017; Rais et al., 2017).

The microbial diversity differed based on plant tissue. The highest diversity was isolated from *T. vulgaris* roots. The diversity of endophytes from the two locations was similar but slightly higher at the Sheikh Zuweid site. It seems that endophytic bacteria isolated from arid land with very low soil nutrient content depend on the type of symbiosis interaction and availability of nutrients in the plant tissue, as has been reported previously (Li et al., 2012; Jin et al., 2014; Liu et al., 2016; Liu et al., 2017; Li et al., 2018).

A major objective of this study was to better understand the interactions between beneficial endophytic bacteria and medicinal plants in arid lands. Therefore, we screened the endophyte collection for beneficial plant traits *in vitro* with a goal of detecting the most promising microorganisms. Many of the endophytes produced several plants promoting traits, in agreement with our previous investigations dealing with medicinal plants in arid lands (Liu et al., 2016; Liu et al., 2017; Li et al., 2018). In addition, similar investigations reported that endophytic bacteria exhibited multiple plant beneficial traits (Egamberdieva and Kucharova, 2009; Egamberdieva et al., 2017b; Paul and Sinha, 2017).

To support the *in vitro* results, three bacterial strains, *Bacillus sonorensis* (EGY05), *Bacillus tequilensis* (EGY21), and *Bacillus mojavensis* (EGY25), were selected for plant growth stimulation properties in a pot experiment under salt stress. Each strain significantly promoted tomato plants at different salt concentrations, compared to un-inoculated controls (**Figure 3**), but each endophyte had a unique pattern of plant growth promotion. For example, *B. sonorensis* EGY05 had the strongest stimulatory effects on roots, but *B. mojavensis* EGY25 had the strongest effects on shoots. These activities are likely due to one more activities, such as nitrogen fixation (Zakry et al., 2012), IAA production (Leveau and Lindow, 2005), or phosphate solubilization (Hameeda et al., 2008; Lemanceau et al., 2009). Additionally, *Bacillus* is particularly resistant to environmental stresses that are common in the desert, such as UV, heat, high alkalinity, and high salinity (Horikoshi, 2008). Several other investigations have also reported that *Bacillus* strains belonging to *Bacillus megaterium* and *Bacillus insolitus* enhanced the length and biomass of shoot, roots, and leaves under salt stress (Ashraf et al., 2004; Radhakrishnan and Lee, 2016; He et al., 2018).

Under conditions of high salinity, plants face disorders in many metabolic pathways, such as those related to redox system and photosynthesis, which leads to reductions in plant growth (Munns and Tester, 2008). To understand the mechanism of plant-microbe responses defense under saline conditions, we investigated the antioxidant enzymes (SOD, CAT, and POD) activity of tomato plants under salt stress. The selected endophytes decreased SOD, POD, and CAT activities at different salt concentrations, compared to the uninoculated controls (**Figure 5**). Enhancement of plant growth during salt stress may be related to increases in the relative water content as well as the osmotic and turgor potential to improve plant growth under salinity conditions (Hashem et al., 2016; Yang et al., 2016). For example, *Bacillus pumilus* reduced the activity of caspase of salt-stressed rice plants (Jha and Subramanian, 2014). However, decreases in the activities of antioxidant enzymes could be due to a general decrease in biosynthesis and lower oxidative stress, as has been suggested by others (Egamberdieva et al., 2017b; Rais et al., 2017). In this study, the total chlorophyll in tomato leaves was slightly increased at different levels of salt concentrations when the plants were subjected to endophytes, but most of these effects were not statistically significant (**Figure 5D**). The reduction in the pigment content is attributed to the negative effect of salt stress on chloroplasts (Ahmad et al., 2012c; Ebrahim and Saleem, 2017).

Tomato (*S. lycopersicum* L.) is one of the most important and widely grown plants in the world. The control of *F. oxysporum* disease has been based almost solely on the application of chemical fungicides. However, for the management of plant diseases, recent efforts have focused on alternative methods of control to circumvent this situation and to develop environmentally friendly and sustainable procedures to control tomato *Fusarium* wilt disease. Thus, one objective of this study was to assess the biological control activities of the endophytes on *Fusarium in vitro* and *vivo.* Our results suggest that antibiotics produced by *Bacillus* endophytes are active against tested phytopathogens. *Bacillus* is well known for its diverse range of secondary metabolic products, including antibiotics (Ryan et al., 2008; Radhakrishnan et al., 2017; Mohamad et al., 2018). Interestingly, all six strains evaluated here could suppress *Fusarium* wilt disease at different levels of salt concentrations, compared to un-inoculated controls (**Figure 6**). Indeed, strain EGY16 was able to reduce disease severity up 26% at all tested salt concentrations. Other investigations reported that *Bacillus* spp. suppress pathogen growth and protect olive and pepper plants (Krid et al., 2012; Yi et al., 2013). In addition, strain EGY16 showed a proteolytic, cellulolytic, and chitinolytic activity that may involve attachment to the mycelial cell walls. These results are in general agreement with (Egamberdieva et al., 2017b) who reported that endophytic bacteria associated with the medicinal plant *Ziziphora capital* were able to produce chitinolytic enzymes. In accordance with these results, several previous studies have shown that endophytic *Bacillus* species control fungal pathogens, including *B. mojavensis* (Bacon et al., 2005), *B. velezensis* (Gao et al., 2017), *B. megaterium* (Gao et al., 2017), and *B. atrophaeus* (Mohamad et al., 2018). *Bacillus* spores are well-known for their ability to survive for a long time under unfavorable environmental conditions. *Bacillus* vegetative cells are able to secrete several metabolites to alleviate abiotic and biotic stresses with antifungal activities and improve plant growth (Chowdhury et al., 2015; Radhakrishnan et al., 2017). In recent years, the development of biological control agents derived from *Bacillus* isolates, such as “Custom” (Baugh and Escobar, 2007), “Avogreen” (Janisiewicz and Korsten, 2002) and “Shemer” (Droby, 2005), has been shown to be eﬀective in plant fungal disease.

In this investigation, among all tested endophytes *in vivo*, *B. subtilis* EGY16 was selected for exometabolomic studies by GC-MS, based on its ability to decrease the disease severity index (DSI) and suppress the growth of *F. oxysporum.* 20 compounds were identified by GC-MS. Among these compounds, five of them were major peaks in cell-free extracts from the co-culture, suggesting that they very likely play a role in antagonism of fungal pathogens. These compounds are known as an antimicrobial compound such as benzene, 1,3-dimethyl-, p-xylene (Fernandes et al., 2003), dibutyl phthalate (Khatiwora et al., 2012), bis(2-Ethylhexyl) phthalate (Kavitha et al., 2009), and tetracosane (Naragani et al., 2016). In addition, several putative antimicrobial compounds were identified as minor peaks: phenylethyl alcohol (Strobel et al., 2001), heneicosane (Sharma et al., 2016), nonadecane (Hsouna et al., 2011), bis(2-Ethylhexyl) phthalate (Kavitha et al., 2009). These results suggesting that these compounds play an important role in antimicrobial activities.

## Conclusion

Globally, crop productivity is decreasing due to climatic change, and human populations are increasing daily. The Food and Agricultural Organization (FAO) predicts an increasing human population to reach 8–9 billion by 2030. Here, we showed that *Bacillus* spp. associated with *T. vulgaris* such as *Bacillus sonorensis* (EGY05), *Bacillus tequilensis* (EGY21), and *Bacillus mojavensis* (EGY25) produced plant growth-promoting substances including auxin, fixed nitrogen, solubilized phosphate and iron, and produced lytic enzymes (i.e., chitinase, cellulase, protease, and lipase). These bacteria may provide new strategies to mitigate salt stress and also develop new ways to enhance the tolerance and growth of plants such as tomato. In addition, all tested strains decreased the activities of antioxidant enzymes (SOD, CAT, and POD) of tomato plants at different salt concentrations, compared to the un-inoculated controls. The selected antagonistic isolates such as *Bacillus sonorensis* (EGY05), *Bacillus subtilis subsp. subtilis* (EGY01), *Bacillus tequilensis* (EGY21), *Bacillus mojavensis* (EGY25), *Enterobacter xiangfangensis* (EGY31), and *Bacillus subtilis subsp. inaquosorum* (EGY16) were able to control tomato root rot significantly (P < 0.05) caused by *F. oxysporum* under greenhouse conditions, suggesting they could be powerful biological control agents. To the best of our knowledge, this is the first study of the diversity of the microbial community associated with medicinal plant *T. vulgaris* and their plant-growth promoting and biocontrol abilities. Further field investigations are ongoing for future application in tomato plant growth promotion and crop productivity as well as antifungal activities against *Fulvia fulva* (Cooke) Cif, and *Alternaria solani* Soraue. These results support the development of natural products that may minimize the need for the application of chemical fertilizer and fungicides, which would be an environmentally friendly approach and preserve biological resources in a sustainable agricultural system.

## Data Availability Statement

Publicly available datasets were analyzed in this study. This data can be found here: GenBank under Accession Numbers MH764457–MH764573.

## Author Contributions

OAAM participated in the design of the study, performed all experiments, interpretation of results and wrote the manuscript. LL participated in the isolation and identification of endophytic bacteria. Y-HL helping for preparing enzymes activity test, and screening for plant beneficial traits. J-BM and DZ conducted the plant growth chamber experiments and data analysis. SH did the GC-MS and data analysis. SB did statistical analysis. BH revised and improved the manuscript. W-JL and OM revised the manuscript and supervised all experiments. All authors edited and critically revised the manuscript.

## Funding

This research was supported by the National Key Research and Development Project (2016YFC0501502), National Natural Science Foundation of China (Grant No. U1703106 and 31650110479), Xinjiang Uygur Autonomous Region Regional Coordinated Innovation Project (Shanghai Cooperation Organization Science and Technology Partnership Program) (Grant No. 2017E01031). OAAM was supported by the Available Position Talented Young Scientists Program funded by Chinese Academy of Sciences President’s International Fellowship Initiative (Grant No. 2016PB024).

## Conflict of Interest

The authors declare that the research was conducted in the absence of any commercial or financial relationships that could be construed as a potential conflict of interest.

## Supplementary Material

Figure S1 A summary of species present at all sites of 117 culturable endophytes from *Thymus vulgaris*. based on 16S rRNA gene sequences.

Figure S2 Distribution of endophytic isolates and species isolated from *Thymus vulgaris* on different media.

Figure S3 Confirmation of plant growth promotion traits by a color change or halo zone on the selective medium (A: Protease; B: Cellulase; C: Lipase; D: Siderophore; E: Chitin; F: Nitrogen). (G) response of endophytic strains to *F. oxysporum* (H) response of endophytic strains to *F. fulva*, (I) response of endophytic strains to *A. solani.*

Figure S4 Effect of endophytic isolates on the growth of tomato (*Solanum lycopersicum* L.) compared with an uninoculated control without salt (CK^+^) and inoculated control with salt (CK^-^).

Figure S5 Effect of inculation of endophytic strains on the root of tomato plants under salt stress.

Table S1 Compositions of the different media used for the isolation of endophytic bacteria from *Thymus vulgaris.*

Table S2 Fungal pathogens used in this study.

Table S3 GC-MS identified components of the antibiosis crude extract of EGY16 and *F. oxysporum* mixture at pH7. (Volatile compounds are listed in ascending order of Retention Time).
