# Supplementary figures and images for: Beneficial Endophytic Bacterial Populations Associated With Medicinal Plant Thymus vulgaris Alleviate Salt Stress and Confer Resistance to Fusarium oxysporum
Source: Front Plant Sci. 2020 Feb 14;11:47. doi: 10.3389/fpls.2020.00047 (PMC7033553; doi:10.3389/fpls.2020.00047)

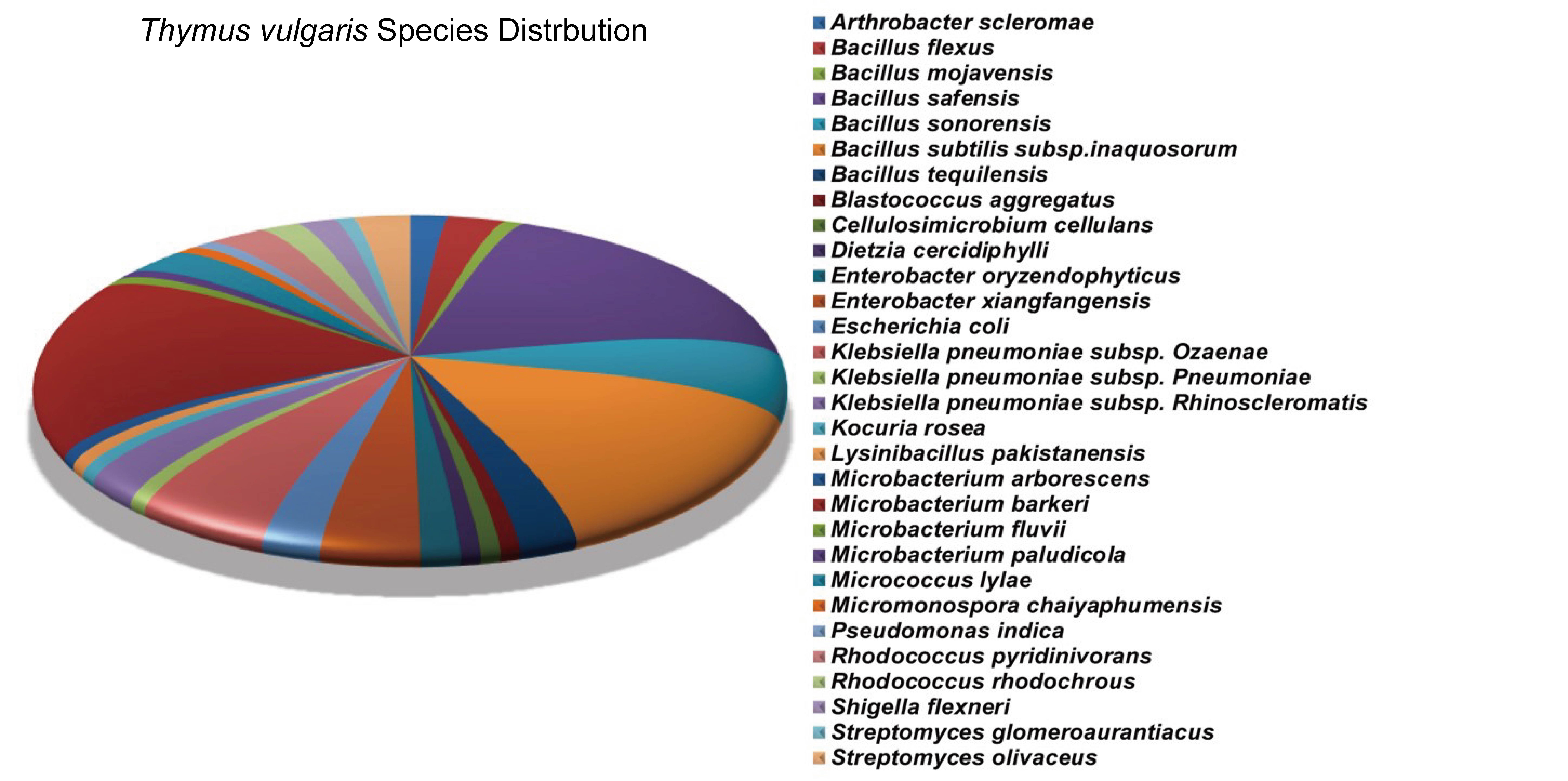

Supplement: Figure S1 — A summary of species present at all sites of 117 culturable endophytes from Thymus vulgaris. based on 16S rRNA gene sequences. [file Image_1.jpeg]

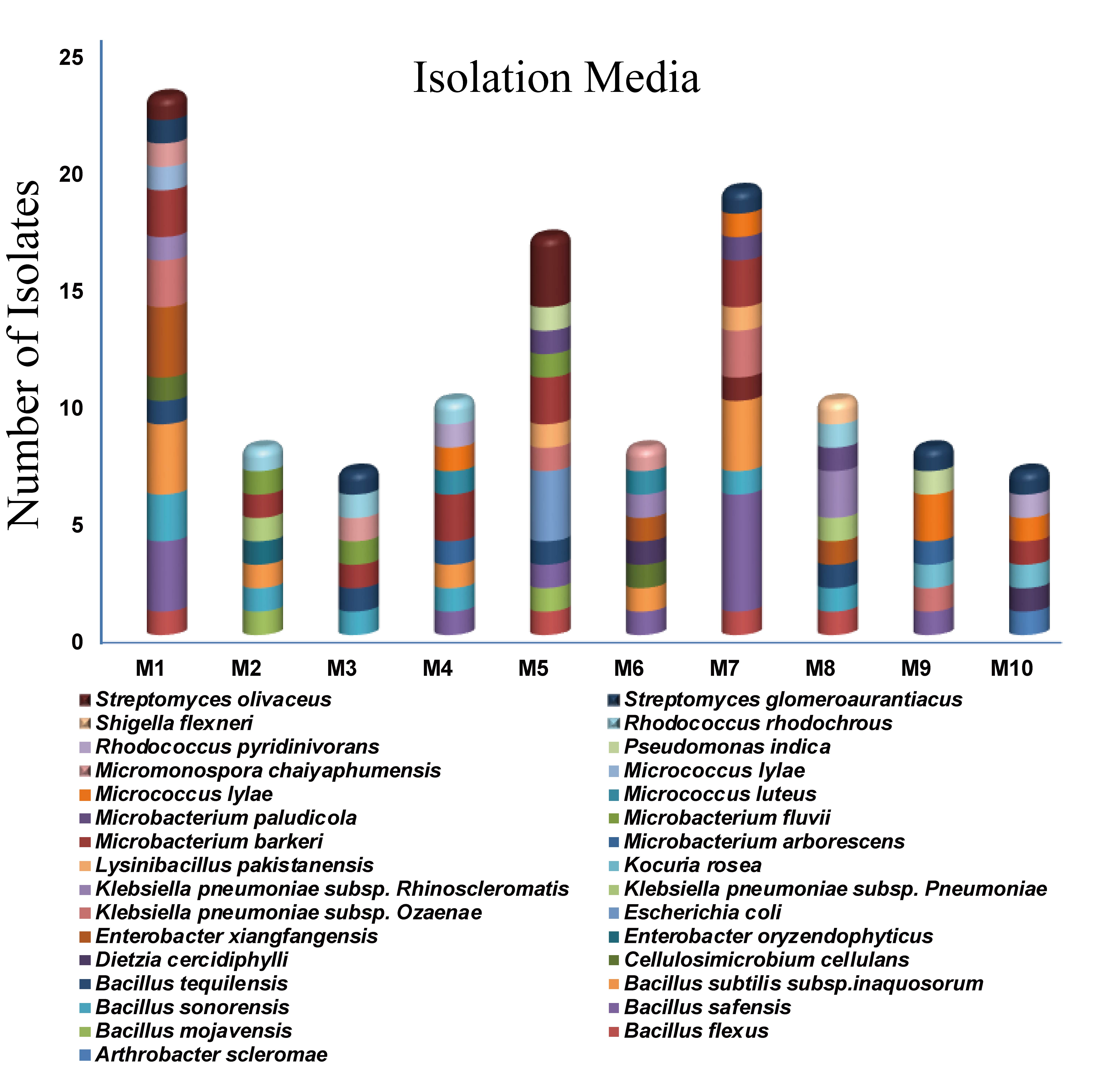

Supplement: Figure S2 — Distribution of endophytic isolates and species isolated from Thymus vulgaris on different media. [file Image_2.jpeg]
