# Supplementary material for: Beneficial Endophytic Bacterial Populations Associated With Medicinal Plant Thymus vulgaris Alleviate Salt Stress and Confer Resistance to Fusarium oxysporum
Source: Front Plant Sci. 2020 Feb 14;11:47. doi: 10.3389/fpls.2020.00047 (PMC7033553; doi:10.3389/fpls.2020.00047)
Supplement: Table S1 — Compositions of the different media used for the isolation of endophytic bacteria from Thymus vulgaris. [file Table_1.doc]

**Table S1.** Compositions of the different media used for the isolation of endophytic bacteria from *Thymus vulgaris*

| **Medium** | **Composition (g/L)** |
| --- | --- |
| M1 | Yeast 0.25g, K_2_HPO_4_ 0.5g, agar 15g. |
| M2 | Trehalose 6g, Proline 1g, KNO_3_ 0.5g, Na_2_HPO_4_ 0.3g, MgSO_4_·7H_2_O 0.2g, CaCl_2_ 0.5g, agar 15g. |
| M3 | Raffinose 5g, L-histidine 1g, KNO_3_ 1g, NaCl 1g, CaCl_2_ 2g, K_2_HPO_4_ 1g, MgSO_4_·7H_2_O 1g, agar 15g. |
| M4 | Sodium propionate 2g, Arginine 1g, NH_4_NO_3_ 0.1g, KCl 0.1g, MgSO_4_·7H_2_O 0.05g, FeSO_4_·7H_2_O 0.05g, agar 15g. |
| M5 | Cellulose 2.5g, Sodium pyruvate 2g, Proline 1g, KNO_3_ 0.25g, MgSO_4_·7H_2_O 0.2g, K_2_HPO_4_ 0.2g, CaCl_2_ 0.5g, FeSO_4_·7H_2_O 0.01g, agar 15g. |
| M6 | Glycerol 10g, Asparagine 1g, K_2_HPO_4_ 1g, trace salt 1mL, agar 15g. |
| M7 | Sodium succinate 1g, L-asparagine 0.2g, KH_2_PO_4_ 0.9g, K_2_HPO_4_ 0.6g, MgSO_4_·7H_2_O 0.1g, CaCl_2_·2H_2_O 0.2g, KCl 0.3g, FeSO_4_·7H_2_O 0.001g, agar 15g. |
| M8 | Dulcitol 2g, Proline 0.5g, K_2_HPO_4_ 0.3g, NaCl 0.3g, MgSO_4_·7H_2_O 1g, CaCl_2_·2H_2_O 1g, agar 15g. |
| M9 | Sodium propionate 2g, L-asparagine 1g, NH_4_NO_3_ 0.1g, KCl 0.1g, MgSO_4_·7H_2_O 0.05g, FeSO_4_·7H_2_O 0.05g, agar 15g. |
| M10 | Yeast 0.3g, Casein 0.3g, Glycose 0.3g, K_2_HPO_4_ 2g, agar 15g. |
| ISP2 | Peptone 5g, Yeast extract 3g, Malt extract 3g, Dextrose 10g, Agar 15g |
