# Supplementary material for: Beneficial Endophytic Bacterial Populations Associated With Medicinal Plant Thymus vulgaris Alleviate Salt Stress and Confer Resistance to Fusarium oxysporum
Source: Front Plant Sci. 2020 Feb 14;11:47. doi: 10.3389/fpls.2020.00047 (PMC7033553; doi:10.3389/fpls.2020.00047)
Supplement: Table S2 — Fungal pathogens used in this study. [file Table_2.doc]

**Table S2:** Fungal pathogens used in this study.

| **Strain** | **Scientific name** | **Host Plant and disease** | **Source** |
| --- | --- | --- | --- |
| F1 | *Fusarium oxysporum* f. sp*.* | Tomato, *Fusarium* wilt | Xinjiang |
| F2 | *Fulvia fulva* (Cooke) Cif. | Tomato, Leaf mildew | Xinjiang |
| F3 | | *Alternaria solani* Sorauer | | --- | | | Tomato, Early Blight | | --- | | | Xinjiang | | --- | |
