# Supplementary material for: Beneficial Endophytic Bacterial Populations Associated With Medicinal Plant Thymus vulgaris Alleviate Salt Stress and Confer Resistance to Fusarium oxysporum
Source: Front Plant Sci. 2020 Feb 14;11:47. doi: 10.3389/fpls.2020.00047 (PMC7033553; doi:10.3389/fpls.2020.00047)
Supplement: Table S3 — GC-MS identified components of the antibiosis crude extract of EGY16 and F. oxysporum mixture at pH7. (Volatile compounds are listed in ascending order of Retention Time). [file Table_3.doc]

**Table. S3:** **GC-MS identified components of the antibiosis crude extract of EGY16 and *F. oxysporum* mixture at pH7.** **(****Volatile compounds are listed in ascending order of Retention Time).**

| **NO** | **Retention Time (min)** | **Compounds** | **Percentage**  **Match %** | **Molecular formula** | **Molecular**  **Weight** |
| --- | --- | --- | --- | --- | --- |
| 1 | 3.444 | Acetic acid, butyl ester | 72 | C_6_H_12_O_2_ | 116.1 |
| 2 | 3.920 | 2,2-Dimethylthiirane | 47 | C_4_H_8_S | 88.171 |
| 3 | 4.055 | 2-Methylpropanoic acid, TMS derivative | 90 | C_7_H_16_O_2_Si | 160.2862 |
| 4 | 4.387 | Benzene, 1,3-dimethyl- | 97 | C_8_H_10_ | 106.168 |
| 5 | 4.582 | Benzene, 1,3-dimethyl- | 97 | C_8_H_10_ | 106.168 |
| 6 | 5.134 | p-Xylene | 97 | C_8_H_10_ | 106.168 |
| 7 | 7.131 | Propane, 1-ethoxy- | 47 | C_5_H_12_O | 88.1482 |
| 8 | 7.309 | Propane, 1-ethoxy- | 38 | C_5_H_12_O | 88.1482 |
| 9 | 11.149 | Hexane-1,3,4-triol, 3,5-dimethyl- | 33 | C_6_H_14_O_3_ | 134.175 |
| 10 | 13.426 | Phenylethyl Alcohol | 97 | C_8_H_10_O | 122.167 |
| 11 | 46.345 | Dibutyl phthalate | 97 | C_16_H_22_O_4_ | 278.348 |
| 12 | 51.961 | Heneicosane | 97 | C_21_H_44_ | 296.5 |
| 13 | 54.866 | Nonadecane | 97 | C_19_H_40_ | 268.529 |
| 14 | 57.652 | Tricosane | 93 | C_23_H_48_ | 324.637 |
| 15 | 60.311 | Tetracosane | 98 | C_24_H_50_ | 338.664 |
| 16 | 62.894 | Bis(2-ethylhexyl) phthalate | 91 | C_24_H_38_O_4_ | 390.564 |
| 17 | 69.486 | Decanedioic acid, bis 2-ethylhex | 64 | C_26_H_50_O_4_ | 426.6728 |
| 18 | 76.215 | Propenone, 1-(3-bromophenyl)-3- | 96 | C_14_H_10_BrNOS | 320.204 |
| 19 | 77.226 | Heneicosane, 11-cyclopentyl- | 47 | C26H52 | 364.702 |
| 20 | 78.211 | cyclohexanecarboxylic acid, 2- | 58 | C_7_H_12_O_2_ | 128.1690 |
